# Kawasaki disease shock syndrome complicated with bilateral lung consolidation in a child: A case report

**DOI:** 10.1097/MD.0000000000020998

**Published:** 2020-07-17

**Authors:** Yue Song, Wuran Wei, Lan Liu, Yibing Wang, Xiaoqing Shi, Li Li

**Affiliations:** a Department of Pediatrics, West China Second University Hospital, Sichuan University, Chengdu, China; b Key Laboratory of Birth Defects and Related Diseases of Women and Children (Sichuan University), Ministry of Education; c Institute of Urology, Department of Urology, West China Hospital, Sichuan University, Chengdu, Sichuan, China.

**Keywords:** Kawasaki disease, Kawasaki disease shock syndrome, lung consolidation

## Abstract

**Introduction:**

Kawasaki disease (KD) is a systemic inflammatory disease. Standard imaging features of KD include interstitial and lobular inflammatory lesions in the lungs, while KD shock syndrome (KDSS), complicated with substantial consolidation and atelectasis in the lung, is rarely reported.

**Patients concerns:**

Herein, we report a single case of a 5-year-old female patient who manifested KDSS on the seventh day of the course of KD. Chest enhanced computed tomography indicated large-area consolidation in the lower lobes of the bilateral lungs.

**Diagnosis:**

The patient was diagnosed with KDSS complicated with non-infective lung consolidation.

**Interventions:**

The patient received human intravenous immunoglobulin (2 g/kg) and aspirin (30–50 mg/kg^.^d), methylprednisolone, a vasoactive agent, and albumin. Infective factors were excluded.

**Outcomes:**

The consolidation in the lower lobe of the bilateral lungs was completely recovered after 3 days of treatment.

**Conclusions:**

Children with KDSS may present with pulmonary lesions such as substantial consolidation and atelectasis; thus, infective factors should be excluded. If there is no etiological evidence, antibiotics should be used with caution.

## 1 Introduction

Kawasaki disease (KD), also known as mucocutaneous lymph node syndrome, is characterized by unclear etiology and acute self-limited and systemic vasculitis, and mainly occurs in children younger than 5 years of age.^[1]^ Except for coronary artery lesions, children with KD can also suffer from injury to multiple systemic systems.^[2–7]^

Kawasaki disease shock syndrome (KDSS) is a severe form of KD that mainly presents with hemodynamic instability during the course of disease, and shock at an early stage. Nevertheless, it is challenging to diagnose KDSS early on. In addition, KDSS with the primary manifestation of acute non-infective pulmonary consolidation rarely occurs.^[8]^

Herein, we report a single case of a patient with KDSS aggravated by noninfective considerable pulmonary consolidation. We analyzed the patient's clinical manifestations, diagnosis, and treatment process.

## 2 Case presentation

A girl aged 5 years and 8 months visited the local hospital because of a mass on the right side of her neck that was present for 5 days, pyrexia that lasted for 4 days, and abdominal pain lasting for 2 days. After receiving treatment with antibiotics, the above symptoms did not improve, and she was admitted to our hospital on the sixth day of the course of the disease. Follow-up echocardiography revealed no coronary artery lesions. Physical examination at admission revealed the following: temperature, 36.8°C; respiration, 30 times/min; heart rate, 85 times/min; and blood pressure, 93/50 mm Hg. Pink papules were observed on the face, trunk, and limbs. Several enlarged lymph nodes were palpable in the bilateral neck (maximum size on the right, 3 × 4 cm; maximum size on the left, 2 × 1 cm) with tough texture, acceptable mobility, clear boundaries with surrounding tissues, and without haphalgesia. Conjunctival congestion was observed in both eyes, rhagades were observed on the lips, and the tongue was strawberry-like. The neck was soft, and cardiac and pulmonary examinations were not remarkable. The abdomen was soft. Upon applying pressure on the right and left lower abdomen, the patient experienced pain. There was no muscular tension or rebound tenderness, the liver and spleen were normal, and the bowel sounds were regular. No swelling of the fingertips was observed, and there was no desquamation in the crissum. The capillary refill time was 2 second. Auxiliary examination showed the following: routine blood examination: white blood cells(WBC), 18.4 × 10^9^/L;N; 86.5%; hemoglobin, 121 g/L; and platelets 293 × 10^9^/L. C-reactive protein (CRP) level was 158 mg/L. Routine urine examination revealed urine protein 1+, ketone body 1+, and bilirubin 3+. Coagulation function test revealed antithrombin-III, 69%; D-dimer, 2.04 mg/L, and fibrin degradation product, 8.20 μg/mL. No abnormalities were observed in renal liver function, electrolytes, amylases, lipases, troponin I (cTn-I), myohemoglobin, or electrocardiogram. Appendicitis color Doppler ultrasound suggested no expansion of the vermiform appendix cavity. Echocardiography indicated normal left ventricular systolic function. A chest radiograph showed a few patchy shadows in the bilateral lungs.

Based on these manifestations (rash, lymphadenectasis in the bilateral neck, conjunctival congestion in both eyes, cracked lips, strawberry-like tongue, hard edema at the end of the fingers and toes, CRP of 158 mg/L, urine protein 1+, and hypercoagulability of coagulation function), KD was diagnosed. The patient was administered intravenous immunoglobulin (IVIG) (2 g/kg) and aspirin (50 mg/kg·d). On the seventh day of the course of disease, the condition progressively aggravated, with fever, general depression edema, increased heart rate, progressive decrease in blood pressure (with the lowest blood pressure measured as 70/30 mm Hg), cold limbs, and obvious hard edema at the end of the fingers and toes, and the capillary refill time  > 3 second observed. Quick examination showed albumin level at 20.9 g/L, Na^+^ at 122.5 mmol/L, cTn-I at 0.190 μg/L, and N-terminal pro-natriuretic peptide at 2463.72 pg/mL. Echocardiography suggested mild to moderate mitral regurgitation, moderate to severe tricuspid regurgitation, mild aortic regurgitation, trace hydropericardium, left coronary artery  = 3.7 mm, and normal left ventricular systolic function; hence, KDSS was considered.

**Figure 1:**
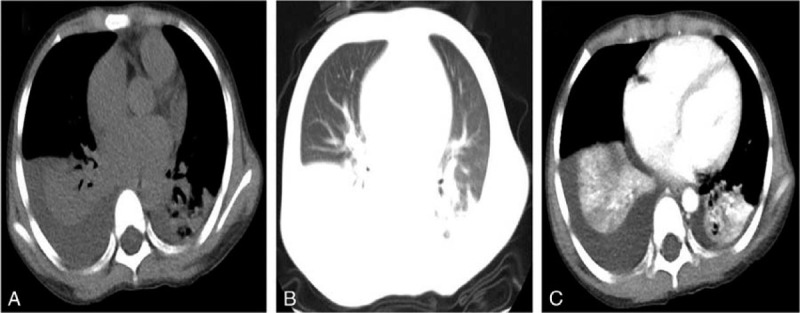
(A-C) Enhanced-chest computed tomography (CT) revealed scattered strip-shadows, and ground-glass shadows. Sub-pulmonary lobes presented with consolidation with atelectasis. In addition, there was a right middle volume of pleural effusion and a small amount of pleural effusion on the left side.

**Figure 2:**
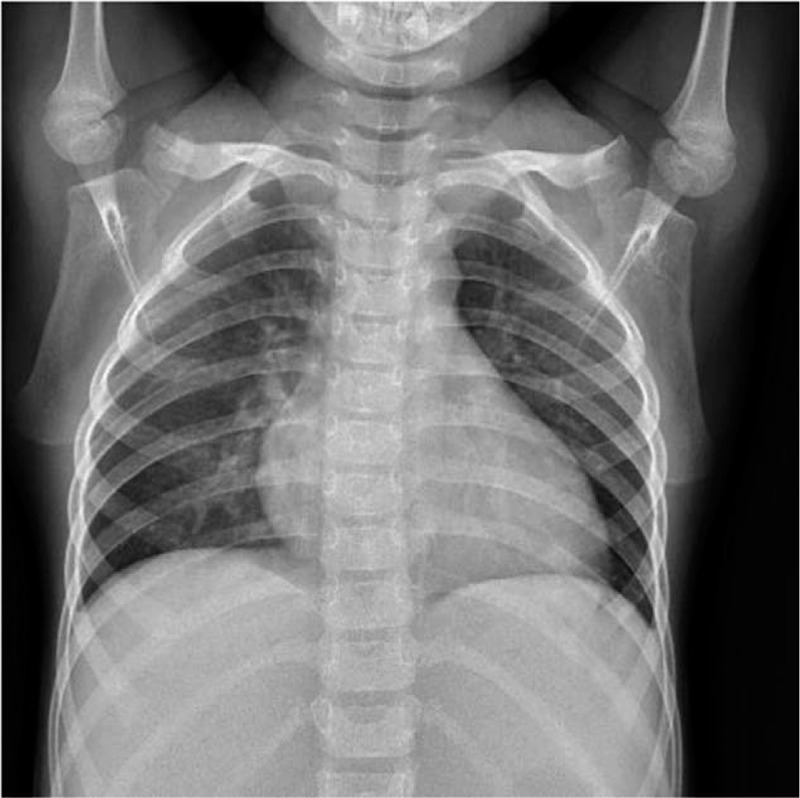
In the absence of antibiotic treatment, consolidation with atelectasis in the lower lobe of the bilateral lungs disappeared on the third day of the course of the disease.

**Figure 3:**
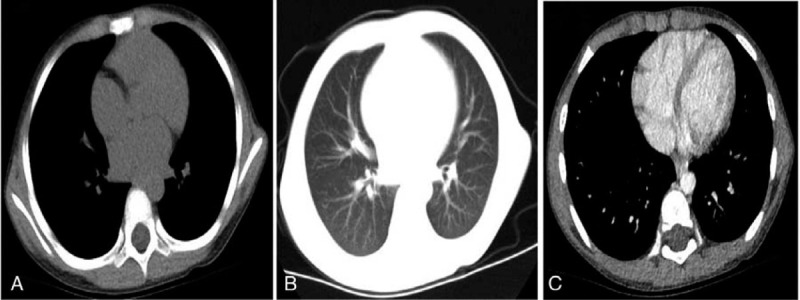
**(**A-C**)** Following 1 wk of treatment, chest enhanced computed tomography (CT) revealed a small sulcus in the basal segment of the left lower lobe; inflammation in the lung was completely absorbed.

After the patient was given oxygen, temporary pumping of noradrenaline (0.01 μg/kg·min), methylprednisolone (25 mg/kg·d), and albumin infusion (3 times, totaling 30 g), her temperature returned to normal, and shock-related manifestations disappeared. However, dyspnea appeared. Chest and abdomen enhanced computed tomography (CT) were immediately performed, indicating large consolidation complicated with atelectasis in the lower lobe, a small amount of pleural effusion in the left lobe, a moderate amount of pleural effusion in the right lobe, thickened and swollen right gall bladder wall, intrahepatic cholestasis, and pelvic effusion (Fig. 1). One hundred ml of hydrothorax was collected and analyzed, indicating transudate. The results of mycoplasma/chlamydia antibody test, EB virus nucleic acid test, sputum culture, hydrothorax culture, and blood culture were all negative. According to the clinical manifestation, features, and laboratory examination results, the large lung consolidation that appeared within a few days was determined to be a manifestation of KD in the lung, rather than toxic shock syndrome (TSS) complicated by a pulmonary bacterial infection. The patient then received a second IVIG shock treatment, after which she was given a small dose of glucocorticoids for anti-inflammation and a large amount of aspirin for 3 days to prevent thrombosis and for anti-inflammation. Reexamination of the blood (performed on 03/30/19) indicated WBC of 25.5 × 10^9^/L, N of 73.6%, and CRP < 0.5 mg/L. Furthermore, chest radiography (performed on 03/30/19) revealed a thickened and dim texture in the bilateral lungs (Fig. 2), while chest enhanced CT (performed on 03/04/19) showed a complete absorption of inflammation in the lung (Fig. 3). Echocardiography (06/04/19) showed moderate tricuspid regurgitation, 3.3 mm left coronary artery, and normal left ventricular systolic function.

## 3 Discussion

Our patient went into shock on the seventh day of the course of the disease (systolic pressure 20% lower than normal for her age), accompanied by elevated neutrophil percentage, CRP, severe hypoproteinemia, hyponatremia, valvular regurgitation, elevated N-terminal pro-natriuretic peptide and cTn-I, coronary artery ectasia, and resistance to IVIG, which suggested a KDSS. After receiving appropriate expansion and vascular activity drugs, a well-timed large dose of IVIG shock, and other supporting treatments, the shock was rapidly controlled. These data were consistent with previous studies reporting outcomes in KDSS.^[9,10]^

During the course of the disease, dyspnea appeared and progressively aggravated. Chest enhanced CT indicated a large consolidation and polyserositis in the lower lobe of the bilateral lungs and increased inflammatory indices. KD can involve multiple systems, and the respiratory system is a commonly involved in children with KD. According to Lee *et al.*, abnormal chest x-ray findings are observed in 51.2% of patients with KD.^[11]^ Common pulmonary manifestations mainly include bronchopneumonia, hydropneumothorax, and pleural effusion. A few studies have reported that KD may appear secondary to lung consolidation, which often occurs due to Streptococcus, Staphylococcaceae, Mycoplasma, EB virus, coronavirus, or parvovirus infection.^[6]^ Nevertheless, there are no reports on respiratory system lesions (non-infective large lung consolidation and atelectasis) in children with KD. Thus, in the current case, it was essential to identify whether lung consolidation and atelectasis appeared secondary to KDSS or TSS.

Blood cultures were consistently negative. Hydrothorax indicated that transudate did not contain bacterial infection. Moreover, lung consolidation and atelectasis appeared during KDSS progression. Thus, pathological changes observed in the lung were considered to be related to KD. In addition, according to the principles of KD therapy without antibiotics, consolidation in the lower lobe was completely absorbed within 3 days, and the course of treatment was much shorter than the cure course of consolidation in the bilateral lungs caused by a bacterial infection, which further confirmed the diagnosis of KD-related pulmonary changes. This suggests that the appearance of KDSS is significant in determining KD severity, and severe pathological changes in other organs should be considered in these patients.

Early manifestations of KDSS are similar to those of TSS; however, the treatment is different. Though fluid resuscitation and maintenance of hemodynamic stability are similar, specific antibiotics and glucocorticoid are used to treat TSS, which still leads to high mortality rates (up to 44%) after treatment.^[12]^ For KDSS, IVIG should be administered as soon as possible to achieve a good prognosis, while the use of antibiotics is not required. When unusual pulmonary changes similar to bacterial infection appear, KDSS and TSS should be identified based on monism, rather than using antibiotics.

KDSS results from a combination of several factors. Possible mechanisms of KDSS include continuous capillary leakage,^[13]^ cardiac dysfunction,^[14]^ and abnormal cytokine regulation.^[9]^ Our patient had light respiratory tract symptoms and imaging manifestations prior to the appearance of KDSS, and showed corresponding progress of KDSS with manifestation in the respiratory system. Based on previous studies on KD, possible mechanisms may include the following:

(1) the patient suffered from KD twice, suggesting that the patient had a genetic predisposition. In addition, the systemic vasculitic response was more evident than in other children.
(2) KDSS resulted from a combination of several factors. When vascular endothelial growth factor (VEGF) is highly expressed, vascular permeability is enhanced, and serum albumin is reduced, leading to a large amount of VEGF permeating from the alveolus and pulmonary interstitium, and lung consolidation. An increase in blood capillary leakage causes polyserositis.
(3) The patient had a moderate amount of pleural effusion, which had a compression effect on the adjacent lung tissue.^[15]^

When this occurs, the corresponding lower lobe shifts and is often associated with subsegmental atelectasis, leading to the conclusion that multiple factors cause pulmonary manifestations. Because lung tissues have a strong compensatory function, there is a very low probability of a large consolidation complicated with atelectasis in the lung, which should be further investigated in our patient.

The successful diagnosis and treatment reported in the current study furthers our understanding of KDSS as a rare but severe manifestation of KD. Early diagnosis and treatment of KDSS could reduce complications and shorten the course of the disease. When KDSS appears, lesions in other organs should also be controlled. Children with KD may show respiratory system changes, such as lung consolidation and atelectasis; thus, any symptoms pointing to infection should be carefully considered. When there is no etiologic evidence, antibiotics should be used with caution, and standard and active treatment should be strictly performed following KDSS therapy for good efficacy.

## Acknowledgments

We thank the doctors at the Department of Radiology of West China Second University Hospital for their help with data collection.

## Author contributions

**Conceptualization:** Yue Song, Wuran Wei, Li Li, Yibin Wang and Xiaoqing Shi

**Data curation:** Yue Song, Wuran Wei, Lan Liu, Li Li.

**Writing – original draft:** Yue Song, Li Li.

**Writing – review & editing:** Li Li.
